# Investigating the risk of bone fractures in elderly patients with type 2 diabetes mellitus: a retrospective study

**DOI:** 10.1186/s12902-019-0413-0

**Published:** 2019-07-26

**Authors:** Takeshi Horii, Makiko Iwasawa, Yusuke Kabeya, Jyunichi Shimizu, Koichiro Atsuda

**Affiliations:** 1 Division of Clinical Pharmacy (Pharmacy Practice and Science I) and Research and Education Center for Clinical Pharmacy, Kitasato University School of Pharmacy, 1-15-1 Kitasato, Minami Ward, Sagamihara City, Kanagawa Prefecture 252-0375 Japan; 2 Division of Clinical Pharmacy (Laboratory of Drug Information) and Research and Education Center for Clinical Pharmacy, Kitasato University School of Pharmacy, 1-15-1 Kitasato, Minami Ward, Sagamihara City, Kanagawa Prefecture 252-0375 Japan; 3 Home Medical Care Department, Sowa Hospital, 1752 Oshima, Midori Ward, Sagamihara City, Kanagawa Prefecture 252-0135 Japan; 4 Department of Pharmacy, Tokyo Saiseikai Central Hospital, 1-4-17 Mita, Minato Ward, Tokyo, 108-0073 Japan

**Keywords:** Type 2 diabetes mellitus, Bone fractures, Antihyperglycemic agents

## Abstract

**Background:**

Elderly patients with type 2 diabetes mellitus (T2DM) experience fractures more frequently than elderly individuals without diabetes. Fractures requiring hospitalization greatly affect quality of life, and although elderly patients with T2DM have several risk factors associated with fractures, only a few studies have evaluated these in detail in the Asian population. We conducted a retrospective study of elderly patients with T2DM for evaluating factors associated with fracture risk.

**Methods:**

We conducted a retrospective study using electronic medical records (EMR) of patients aged ≥65 years with T2DM who were admitted to a public general medical institute in central Tokyo, Japan. We evaluated factors associated with fractures necessitating hospitalization in elderly patients with T2DM characteristics and hypoglycemic agent use. Factors associated with fracture risk were identified using multivariable logistic regression analysis.

**Results:**

A total of 2,112 elderly patients (age ≥ 65 years) with T2DM were analyzed. Among them, 69 (3.3%) patients had been hospitalized for fractures. Factors associated with fractures were female sex (OR, 3.46), eGFR < 60 ml / min / 1.73 m^2^ (OR, 0.55), and thiazolidine use (OR, 4.28). Further, a separate analysis based on sex revealed that the use of thiazolidines was significantly associated with fracture risk in both sexes.

**Conclusions:**

In elderly patients with T2DM, the key factor associated with fractures was the use of thiazolidines in both males and females. In this study, the use of thiazolidines was newly identified as a factor which increased the risk of fractures requiring hospitalization in elderly males. The study findings should be considered when hypoglycemic agents are selected for treating elderly patients with T2DM. Information bias, selection bias, and the effect of concomitant drugs may be the underlying reasons for why eGFR < 60 mL / min / 1.73 m^2^ reduced the fracture risk. However, details are unknown, and additional investigations are needed.

## Background

The worldwide prevalence of diabetes has been on the rise, and Japan ranks among the top 10 countries in terms of the number of elderly patients with diabetes. Moreover, the increasing proportion of the elderly in Japan includes the diabetic population. Decreased bone mass and pathological aging put elderly patients at risk for bone fractures, which reportedly negatively affect activities of daily living (ADLs); in challenging and serious cases, such fractures increase the risk of mortality [1–3]. A study on patients aged ≥55 years who were hospitalized for fractures demonstrated that 7.4% of the patients were discharged with medical care services and 53.2% were transferred to another medical facility [4]. Thus, fractures requiring hospitalization for elderly patients have a significant effect on their quality of life (QOL). Patients with type 2 diabetes mellitus (T2DM) are at a higher risk of bone fractures than individuals without diabetes, regardless of bone density [5, 6].

In Japan, the frequently used hypoglycemic agents comprise seven oral drugs and two injectable drugs, acting via nine different mechanisms. Among these agents, the only family of agents that has a clear correlation with risk of fractures is the thiazolidine (TZD) family, which has been found to increase the risk of bone fractures in females [7–10]. Although reports have also implicated other types of drugs in the occurrence of bone fractures [10], no consensus has been reached. Elderly patients with T2DM have various risk factors associated with fractures, but few studies have evaluated these in detail in the Asian population. The aim of the present study was to investigate the relationship between patient background, hypoglycemic agents, and risk factors in elderly patients with T2DM in Japan.

## Methods

### Subjects

We conducted a retrospective study using electronic medical records (EMR) of patients aged ≥65 years with T2DM mellitus who were admitted to Tokyo Saiseikai Central Hospital, which is a public general medical institute in central Tokyo, Japan. Data from January 2014 to January 2016 were used for analyses.

The inclusion criteria were (1) age ≥ 65 years, (2) a diagnosis of T2DM (ICD-10 code E11) or diabetes (ICD-10 code E14), and (3) inpatient status. The exclusion criteria were (1) a diagnosis of type 1 diabetes or no diagnosis of diabetes, (2) age < 65 years, (3) outpatient status, or (4) hospitalized ≥2 times within the study period. Data regarding patient characteristics and medicines used were recorded on the first day of hospitalization.

On the basis of the blood glucose target levels recommended by the Japanese Diabetes guidelines for patients with diabetes, the cutoff value for HbA1c was < 7.0 and that for BMI was < 25 kg/m^2^.

### Survey items

We recorded patient characteristics and the number and type of regular oral medications used by each patient at the time of admission, and the use of hypoglycemic agents including sulfonylureas (SU), glinides, biguanides (BG), TZD, α-glucosidase inhibitors (αGI), sodium glucose cotransporter 2 (SGLT2) inhibitors, dipeptidyl peptidase-4 (DPP-4) inhibitors, glucagon-like peptide-1 (GLP-1) receptor agonist, and insulin.

### Statistical analysis

Patient characteristics were tabulated as mean values plus or minus the standard deviation. Continuous variables were analyzed using unpaired t-test. Nominal variables were compared using χ^2^ test. Logistic regression was used for identifying which factors affect the incidence of fractures, using fractures necessitating hospitalization as the target variable. Univariate analysis was performed using explanatory variables including patient characteristics, hypoglycemic agent usage, and dosing of SU, glinides, BG, TZD, αGI, SGLT2 inhibitors, DPP-4 inhibitors, GLP-1 receptor agonist, and insulin. Explanatory variables with a significance of *p* < 0.2 were extracted using univariate analysis, and odds ratios (ORs) [at 95% confidence interval (CI)] were calculated using multivariate analysis. Because bone fracture incidence differs between males and females [11], the patients were separately analyzed on the basis of sex using the same method as described above for each subgroup. All statistical analyses were performed using the software Stata version 10 (stata corp, TX USA), and the statistical significance threshold was set at < 5%.

Ethics approval: This study was conducted in accordance with ethical guidelines for medical and health research involving human subjects. The ethics board of the Tokyo Saiseikai Central Hospital approved the study (control number, 27–51).

## Results

### Subjects

Patient background characteristics are shown in Table 1. There were a total of 2,112 subjects with a mean age of 76.5 ± 7.1 years, among which 55.3% were aged ≥75 years. Of them, 69 patients (3.3%) experienced fractures that necessitated hospitalization (hip fracture, *n* = 22; humeral fracture, *n* = 9; femoral neck fracture, *n* = 19; and spine fracture, n = 19). The mean HbA1c level was 7.1% ± 1.2%. Hypoglycemic agents were not administered to 33.1% (*n* = 700) of the patients. The most widely used hypoglycemic agents were DPP-4 inhibitors, used in 42.8% (*n* = 904) of patients, followed by SU (25.7%, *n* = 542), insulin (20.8%, *n* = 440), BG (19.3%, *n* = 407), αGI (9.5%, *n* = 200), TZD (7.0%, *n* = 148), GLP-1 receptor agonist (1.8%, *n* = 39), and SGLT2 inhibitors (0.4%, *n* = 8). None of the patients used glinides. We compared their background characteristics by dividing them into the following two groups: fracture group and no fracture group. Significant differences were noted in the following factors: percentage of males (fracture vs. non fracture, 36.8% vs. 65.5%, *p* < 0.001), age (fracture vs. non fracture, 79.7 ± 7.6 years vs. 76.4 ± 7.1 years, *p* = 0.002), the eGFR (fracture vs. non fracture, 62.1 ± 23.9 mL/min/1.73 m^2^ vs. 54.3 ± 22.1 mL/min/1.73 m^2^, *p* = 0.02), and TZD use (fracture vs. non fracture, 15.9% vs. 6.7%, *p* = 0.03).

**Table 1:** Patient characteristics

	All Cases	No fractures	Fractures	*P* value
	*n* = 2112	*n* = 2043	*n* = 69	
Sex (male, %)	64.9	65.6	36.8	< 0.001
Age (years)	76.5 ± 7.1	76.4 ± 7.1	79.7 ± 7.6	0.002
BMI (kg/m^2^)	23.1 ± 4.1	23.1 ± 4.1	22.3 ± 5.0	0.9
HbA1c (%)	7.1 ± 1.2	7.1 ± 1.2	6.8 ± 1.4	0.92
eGFR (mL/min/1.73m^2^)	54.5 ± 22.1	54.3 ± 22.1	62.1 ± 23.9	0.02
Patients with fractures, *n* (%)	69 (3.3%)	0 (0)	69 (100)	< 0.001
Number of drugs being administered at the time of hospitalization	7.7 ± 3.8	7.7 ± 3.8	7.7 ± 3.3	0.52
Patients not using hypoglycemic agent, n (%)	700 (33.1)	674 (33.0)	26 (36.8)	0.62
Glinides, n (%)	0	0	0	–
SGLT2 inhibitors, n (%)	8 (0.4)	8 (0.4)	0	0.7
TZD, n (%)	148 (7.0)	137 (6.7)	11 (15.9)	0.03
αGI, n (%)	200 (9.5)	194 (9.5)	6 (8.7)	0.74
BG, n (%)	407 (19.3)	398 (19.5)	9 (13.0)	0.17
SU, n (%)	542 (25.7)	527 (25.8)	15 (21.7)	0.3
DPP-4 inhibitors, n (%)	904 (42.8)	877 (42.9)	27 (39.1)	0.45
GLP-1 receptor agonists, n (%)	39 (1.8)	36 (1.8)	3 (4.3)	0.11
Insulin, n (%)	440 (20.8)	425 (20.8)	15 (21.7)	0.66

Mean ± S.D., BMI (body mass index), HbA1c (hemoglobin A1c), eGFR (estimated glomerular filtration rate), SGLT2 inhibitors (sodium glucose cotransporter 2 inhibitors), TZD (thiazolidines), αGI (α-glucosidase inhibitors), BG (biguanides), SU (sulfonylureas), DPP-4 inhibitors (dipeptidyl peptidase 4 inhibitors), GLP-1 receptor agonist (glucagon-like peptide-1 receptor agonist)

Table 2 shows patient characteristics stratified by sex. Females had a higher age (78.1 ± 7.6 years vs. 76.4 ± 6.7 years, *p* < 0.001) and higher number of fractures (7.0% vs. 1.2%, *p* < 0.001), and a greater proportion of them were not using hypoglycemic agents (38.4% vs. 30.3%, *p* < 0.001) compared with males. On the contrary, a significantly larger proportion of females were using SU (females, 38.4% vs. males, 30.3%, *p* = 0.001) and a significantly smaller proportion were using TZD (females, 5.3% vs. males, 8.0%, *p* = 0.04) compared with males.

**Table 2:** Male and female patient characteristics

	Males	Females	*P* value
	*n* = 1371	*n* = 741	
Age (years)	76.4 ± 6.7	78.1 ± 7.6	< 0.001
≥ 65–< 75, *n* (%)	680 (49.6)	264 (35.6)	
≥ 75, (*n*, %)	691 (50.4)	477 (64.4)	
BMI (kg/m^2^)	23.1 ± 3.9	23.2 ± 4.6	0.28
< 25, *n* (%)	999 (72.9)	507 (68.4)	
≥ 25, *n* (%)	372 (27.1)	234 (31.6)	
HbA1c (%)	7.1 ± 1.1	7.1 ± 1.4	0.24
< 7%, *n* (%)	714 (52.1)	409 (55.2)	
≥ 7%, *n* (%)	657 (47.9)	332 (44.8)	
eGFR (mL/min/1.73m^2^)	54.3 ± 22.2	54.7 ± 22.0	0.35
Patients with fractures, *n* (%)	17 (1.2)	52 (7.0)	< 0.001
Number of drugs being administered at the time of hospitalization (agents)	7.7 ± 3.9	7.7 ± 3.7	0.4
Patients not using hypoglycemic agent, *n* (%)	415 (30.3)	285 (38.4)	< 0.001
Glinide, *n* (%)	0	0	–
SGLT2 inhibitors, *n* (%)	7 (0.5)	1 (0.1)	0.25
TZD, *n* (%)	109 (8.0)	39 (5.3)	0.04
αGI, *n* (%)	126 (9.2)	74 (10.0)	0.63
BG, *n* (%)	268 (19.5)	139 (18.8)	0.66
SU, *n* (%)	388 (28.3)	154 (20.8)	0.001
DPP-4 inhibitors, *n* (%)	609 (44.4)	295 (39.8)	0.06
GLP-1 receptor agonist, *n* (%)	26 (1.9)	13 (1.8)	0.84
Insulin, *n* (%)	295 (21.4)	145 (19.6)	0.38

Mean ± S.D., BMI (body mass index), HbA1c (hemoglobin A1c), eGFR (estimated glomerular filtration rate), SGLT2 inhibitors (sodium glucose cotransporter 2 inhibitors), TZD (thiazolidines), αGI (α-glucosidase inhibitors), BG (biguanides), SU (sulfonylureas), DPP-4 inhibitors (dipeptidyl peptidase 4 inhibitors), GLP-1 receptor agonist (glucagon-like peptide-1 receptor agonist)

### Factors affecting fracture risk

Results of logistic regression analysis using patient characteristics and hypoglycemic agent use as the explanatory variables and fracture occurrence as the response variable are shown in Table 3. Multivariate analysis with fracture as the objective variable revealed statistically significant differences for female sex (OR, 3.46; 95% CI, 1.88–6.37; p < 0.001), eGFR < 60 mL / min / 1.73 m^2^ (OR, 0.55; 95% CI, 0.31–0.99; *p* = 0.04), and TZD use (OR, 4.28; 95% CI, 1.71–10.7; *p* = 0.002).

**Table 3:** Factors affecting fracture risk

	All patients				Males				Females			
	Univariate analysis		Multivariate analysis		Univariate analysis		Multivariate analysis		Univariate analysis		Multivariate analysis	
	OR (95% CI)	*P* value	OR (95% CI)	*P* value	OR (95% CI)	*P* value	OR (95% CI)	*P* value	OR (95% CI)	*P* value	OR (95% CI)	*P* value
Sex (Ref: M)												
Males	Reference	–	Reference	–								
Females	3.64 (2.01–6.60)	< 0.001	3.46 (1.88–6.37)	< 0.001								
Age (years)												
≥ 65–< 75	Reference	–	Reference	–	Reference	–	Reference	–	Reference	–	–	–
≥ 75	1.98 (1.06–3.70)	0.03	1.63 (0.84–3.15)	0.15	2.32 (0.81–6.63)	0.12	2.23 (0.77–6.41)	0.14	1.34 (0.61–2.94)	0.46	–	–
BMI (kg/m^2^)												
≥ 25	Reference	–	Reference	–	Reference	–	–	–	Reference	–	Reference	–
< 25	1.85 (0.87–3.99)	0.11	1.90 (0.86–4.20)	0.11	1.60 (0.46–5.62)	0.46	–	–	2.16 (0.82–5.69)	0.12	2.35 (0.86–6.43)	0.1
eGFR (mL/min/1.73m^2^)												
≥ 60	Reference	–	Reference	–	Reference	–	–	–	Reference	–	Reference	–
< 60	0.66 (0.37–1.17)	0.15	0.55 (0.31–0.99)	0.04	0.85 (0.31–2.20)	0.73	–	–	0.58 (0.28–1.18)	0.13	0.52 (0.25–1.08)	0.08
HbA1c (%)												
< 7.0	Reference	–	–	–	Reference	–	Reference	–	Reference	–	–	–
7.0–< 8.0	0.88 (0.44–1.77)	0.73	–	–	0.32 (0.07–1.44)	0.14	0.36 (0.08–1.67)	0.19	1.54 (0.68–3.50)	0.30	–	–
≳8.0	0.54 (0.20–1.41)	0.21	–	–	0.81 (0.22–2.92)	0.75	0.98 (0.26–3.70)	0.97	0.34 (0.08–1.49)	0.15		
hypoglycemic agent												
Using	Reference	–	Reference	–	Reference	–	Reference	–	Reference	–	–	–
Not using	1.66 (0.94–2.93)	0.08	1.37 (0.70–2.68)	0.36	2.04 (0.78–5.32)	0.15	2.00 (0.63–6.02)	0.15	1.26 (0.62–2.58)	0.52	–	–
SGLT2 inhibitors	–	–	–	–	–	–	–	–	–	–	–	–
TZD	2.29 (1.01–5.19)	0.04	4.28 (1.71–10.7)	0.002	2.64 (0.75–9.35)	0.13	4.70 (1.14–19.3)	0.03	2.50 (0.83–7.49)	0.83	4.71 (1.43–15.5)	0.01
αGIs	0.61 (0.19–1.98)	0.41	–	–	1.23 (0.28–5.44)	0.79	–	–	0.31 (0.42–2.32)	0.26	–	–
BG	0.38 (0.14–1.07)	0.07	0.47 (0.16–1.43)	0.19	0.26 (0.03–2.00)	0.2	0.37 (0.05–3.06)	0.36	0.46 (0.14–1.52)	0.2	0.53 (0.15–1.91)	0.33
SU	0.55 (0.26–1.18)	0.13	0.66 (0.28–1.56)	0.35	1.06 (0.37–3.04)	0.91	–	–	0.36 (0.11–1.21)	0.1	0.32 (0.10–1.14)	0.08
DPP-4 inhibitors	0.64 (0.35–1.17)	0.15	0.84 (0.38–1.86)	0.67	0.68 (0.25–1.86)	0.46	–	–	0.67 (0.31–1.43)	0.26	–	–
GLP-1 receptor agonists	2.40 (0.56–10.2)	0.24	–	–	3.21 (0.41–25.1)	0.27	–	–	2.24 (0.28–18.0)	0.45	–	–
Insulin	1.06 (0.52–2.13)	0.22	–	–	1.70 (0.59–4.86)	0.32	–	–	0.78 (0.29–2.06)	0.62	–	–

*BMI* (Body mass index), *eGFR* (Estimated glomerular filtration rate), *HbA1c* (Hemoglobin A1c), *SGLT2* inhibitors (sodium glucose cotransporter 2 inhibitors), *TZD* (thiazolidines), αGI (α-glucosidase inhibitors), *BG* (Biguanides), *SU* (Sulfonylureas), DPP-4 inhibitors (dipeptidyl peptidase 4 inhibitors), GLP-1 receptor agonists (glucagon-like peptide-1 receptor agonists)

### Factors affecting the incidence of fractures in both sexes

Multivariate analysis with fracture as the objective variable (Table 3) revealed a statistically significant difference associated with TZD use (males: OR, 4.70; 95% CI, 1.14–19.3; *p* = 0.03, females: OR, 4.71; 95% CI, 1.43–15.5; *p* = 0.01).

## Discussion

The present study found that female sex and TZD use are significant risk factors for bone fractures requiring hospitalization, and that an eGFR of < 60 mL / min / 1.73 m^2^ is associated with reduced fracture risk. Separate analysis of male and female patient characteristics revealed that TZD use is a risk factor for both sexes.

When estrogen levels decrease at menopause (at approximately 50 years of age), there is a significant accompanying decrease in bone mass, which progresses toward bone loss and osteoporosis [12]. Because the mean age of the females in the present study was 78.1 ± 7.6 years, and because 64.4% of the patients were aged ≥75 years, it was assumed that many of the females had gone through menopause approximately 20 years ago, and that their risk of bone fractures (relative to males) had increased via the same mechanism as in non-diabetic patients.

In the present study, an eGFR < 60 mL / min / 1.73 m^2^ was associated with a decreased risk of fractures and an OR of 0.55. Although chronic kidney disease (CKD) can cause bone fragility independent of other factors, the risk of bone fractures reportedly further increased with disease progression as a result of renal osteodystrophy caused by secondary hyperparathyroidism [13]. However, whether decreased kidney function increases the risk of bone fractures remains to be clarified; some researchers have reported that it does [14, 15], while others have reported that it does not [16, 17]. Administering activated vitamin D3 and calcium carbonate to patients with secondary hyperparathyroidism induced by decreased renal function may reduce the risk of fractures, and further investigation of the effects is warranted.

In terms of the relationship between glycemic control state and bone fracture risk, the risk of bone fractures reportedly increase with higher HbA1c levels [18]. Although HbA1c level was stratified and analyzed in this study, a significant reduction in fracture risk was not observed. Because bone turnover markers improve in the short term by controlling blood sugar [19], fracture risk may be correlated with the state of glycemic control. However, the ACCORD BONE ancillary study found that the risk of fractures was not improved in an intensive glycemic control group [20]. Therefore, good glycemic control in the short term may not be sufficient for reducing the risk of fractures. However, there was insufficient data in the present study to show the effect of glycemic control on fracture risk over a fixed duration, because only the HbA1c value targeted for investigation were used and no subsequent observations were made.

Odds ratios obtained in the present study suggest that TZD use significantly increases the odds of fractures in males (OR, 4.70), females (OR, 4.71), and overall (OR, 4.28). The relationship between T2DM use and the risk of fractures is complex, because the risk of fractures is not only affected by complications and patient characteristics, but also by treatments used in glucose level management. Several studies have reported that TZD use increases the risk of fractures in females, especially in postmenopausal females [21, 22]. This provides an explanation for why the rate of TZD use was significantly lower in females than in males. The use of TZD reportedly increases the risk of fracture in males aged ≥50 years with type 2 diabetes [23]. Because the study population involved patients aged ≥65 years, it can be concluded that use of TZD increased the risk of fracture. After 2011, when administration of the TZD pioglitazone was reportedly associated with bladder cancer [24], treatments with TZD were initiated with proper care. Therefore, many of the subjects in the present study were administered TZDs before 2011, and the increased OR in males may have resulted from the long-term TZD administration and because TZDs tend to be administered to patients with a prolonged history of morbidity.

Our study had several important limitations. First, it was a retrospective study conducted at a single institution and using electronic medical records (EHR); therefore, information and selection biases that may have affected results could not be eliminated. Second, patients with bone fractures who did not require hospitalization were excluded; thus, the study population may have been biased toward more severe cases being selected. Third, because TZDs promote weight gain, they were more likely to be administered (in light of their obesogenic properties) to non-overweight, low-BMI patients already at risk of fractures, which may have influenced the increased fracture risk associated with TZD use. Fourth, as this was a cross-sectional study, it was not possible to examine the effect of the length of administration period of diabetes drugs on fracture risk. Finally, because this study did not use data collected for research purposes, it lacks data regarding renal function, such as albuminuria, and information regarding Vitamin D3 and calcium preparations used for advanced renal dysfunction. Therefore, a detailed study stratifying kidney function could not be conducted.

## Conclusions

In elderly patients with T2DM, the key factor associated with fractures was TZD use in both males and females. The present study also showed that TZD use increases risk of bone fractures necessitating hospitalization in males. These results may need to be considered while selecting hypoglycemic agents for treating elderly patients with T2DM. Information bias, selection bias, and the effect of concomitant drugs may be the underlying reasons for why eGFR < 60 mL / min / 1.73 m^2^ reduced the fracture risk. However, details are unknown, and additional investigations are needed.

## Data Availability

The datasets analyzed during the current study are available from the corresponding author on reasonable request.

## Abbreviations

ADLs: activities of daily living
BG: biguanides
BMI: body mass index
CI: confidence interval
CKD: chronic kidney disease
DPP-4: dipeptidyl peptidase-4
eGFR: estimated glomerular filtration rate
GLP-1: glucagon-like peptide-1
HbA1c: hemoglobin A1c
ORs: Odds ratios
QOL: quality of life
SGLT2: sodium glucose cotransporter 2
SU: sulfonylureas
TZD: thiazolidines
αGI: α-glucosidase inhibitors
